# Rugged and Compact Three-Axis Force/Torque Sensor for Wearable Robots

**DOI:** 10.3390/s21082770

**Published:** 2021-04-14

**Authors:** Heeyeon Jeong, Kyungjun Choi, Seong Jun Park, Cheol Hoon Park, Hyouk Ryeol Choi, Uikyum Kim

**Affiliations:** 1 Department of Robot and Mechatronics, Korea Institute of Machinery & Materials (KIMM), 156, Gajeongbuk-ro, Yuseong-gu, Daejeon 34103, Korea; gmldus1493@kimm.re.kr (H.J.); choikj@kimm.re.kr (K.C.); sjpark61@kimm.re.kr (S.J.P.); parkch@kimm.re.kr (C.H.P.); 2 Department of Mechanical Engineering, Sungkyunkwan University, 2066, Seobu-ro, Jangan-gu, Suwon 16419, Korea

**Keywords:** three-axis force/torque sensor, capacitive sensor, wearable sensor, compact and high-load sensor, neural network calibration

## Abstract

In the field of robotics, sensors are crucial in enabling the interaction between robots and their users. To ensure this interaction, sensors mainly measure the user’s strength, and based on this, wearable robots are controlled. In this paper, we propose a novel three-axis force/torque sensor for wearable robots that is compact and has a high load capacity. The bolt and nut combination of the proposed sensor is designed to measure high-load weights, and the simple structure of this combination allows the sensor to be compact and light. Additionally, to measure the three-axis force/torque, we design three capacitance-sensing cells. These cells are arranged in parallel to measure the difference in capacitance between the positive and negative electrodes. From the capacitance change measured by these sensing cells, force/torque information is converted through deep neural network calibration. The sensing point can also be confirmed using the geometric and kinematic relation of the sensor. The proposed sensor is manufactured through a simple and inexpensive process using cheap and simply structured components. The performance of the sensor, such as its repeatability and capacity, is evaluated using several experimental setups. In addition, the sensor is applied to a wearable robot to measure the force of an artificial muscle.

## 1. Introduction

With the recent rapid growth of the wearable robot market, a variety of wearable robots are being developed and utilized for various purposes, such as for the rehabilitation of patients suffering from neurological or musculoskeletal disorders or assisting elderly people suffering from weak muscle strength [1,2,3,4]. Wearable robots also assist workers to prevent musculoskeletal disorders and improve productivity at industrial sites [5,6,7,8,9]. The technology that enables robots to interact with humans so that they can make their own decisions is called human–robot interaction (HRI). In wearable robots, HRI is more important than in other service robots because it is combined with part of the user’s body, such as the arms, legs, and waist. For HRI, sensors applied to wearable robots should be able to measure and process the wearer’s judgment and will in real time. To achieve HRI in wearable robots, a wide variety of information, such as human joint angles, electromyography signals, and applying force, is required. In particular, force measurement enables wearable robots, including various robots, to perform previously difficult tasks. Therefore, by integrating force sensors into wearable robots, such robots can be used as additional human muscles to improve the user’s muscular strength and endurance. The force information makes it possible to ensure safety by monitoring the physical force between the robot and the user or by recognizing human intentions while the robot is operating. Therefore, for the development of advanced wearable robots, the development of force sensors that are applicable to such robots is highly desired.

To date, many force sensors have been developed for application in wearable robots. By using force measurement methods, the force sensors applied to wearable robots are divided into several types: resistive, piezoelectric, and optical types.

Resistive force sensors, which are known as force-sensitive resistors (FSRs), are polymer film devices. Choi et al. [10] proposed a compact force sensor system for a hip-mounted exoskeleton. The FSR is used to measure the interacting assistance force between the exoskeleton and the user’s lower limb. This sensor can measure the normal force and has a compact size. However, owing to the accuracy and insufficient sensing range of this type of sensor, it is difficult to apply it to wearable robots. Additionally, because FSRs can only measure the normal direction force, additional structures are required to measure the multiaxis forces. Among the resistive sensors, those using strain gauges have been developed. Strain gauge-based force sensors consist of flexure elements and a strain gauge. Strain gauge-based sensors can have a wide sensing range as the measurement range varies depending on the elastic limit range of the flexure elements. Grosu et al. [11] developed a multiaxis strain gauge-based force sensor for HRI in a rehabilitation robotic device. This strain gauge-based sensor was designed to measure the pelvic interaction forces in a rehabilitation exoskeleton device. The sensor had a high capacity that could measure approximately 300 N (X-axis and Y-axis) and 2000 N (Z-axis). It was also designed to be strong enough to withstand all interaction forces. However, the size (80 × 96 mm) and weight (340 g) of the sensor are not suitable for application in wearable robots.

Piezoelectric force sensors have characteristics that are similar to those of strain gauge-based sensors. Such sensors use elements with a piezoelectric effect and convert vibrations into electricity and vice versa. Morales et al. [12] proposed a wearable piezoelectric sensor. This file-based sensor can measure high uniaxial compressive forces of up to 50 kN and is significantly thin (11 μm). A sensor with these advantages can be integrated into clothing to gather real-world data. However, because such sensors require additional devices to amplify the large operating voltage and additional components to obtain multiaxis force information, they are not suitable for application in wearable robots.

Optical force sensors measure force by placing an elastic element in the linear section of the optical sensor to measure the displacement of the elastic element. Gu et al. [13] developed a force sensor based on an optical sensor for a wearable robotic system. An inexpensive photo-interrupter sensor is used to measure the displacement of the sensor’s elastic element. Accordingly, the sensor can measure bidirectional forces (compression and tension). The developed sensor has a significantly wide sensing range of approximately 2 kN, compact size, and light weight. However, because this type of sensor needs to be designed using elastic elements that can guarantee large displacements, the stiffness of such sensors is lower, and linearity and hysteresis characteristics are inferior as large displacements occur.

To summarize the pros and cons of each type of sensor, first, the resistive type FSR sensor can be fabricated as thin and lightweight films; however, its accuracy and sensing range are not sufficient to enable its application to wearable robots. The strain-gauge type sensor has high sensitivity and a sufficient sensing range. However, owing to its complex structure, it is expensive and difficult to use. The piezoelectric type sensor has similar advantages to the strain-gauge type sensor. However, an addition amplifier is needed to supply an operating voltage. Finally, the optical type sensor is inexpensive to manufacture and has a wide sensing range. However, owing to its structure, the wider the sensing range, the worse the sensor characteristics.

Looking at the features of the sensors developed for application in wearable robots from the perspective described earlier, force sensors for use in wearable robotic systems should fulfill the following requirements. First, the sensor should demonstrate a high sensing performance. It is important to develop a high-performance force sensor, and because the performance is high, intention recognition and safety can be secured more reliably and effectively. Here, a high performance of the sensor means an appropriate force-sensing range and high sensitivity. Generally, a human’s vertebrate muscle typically produces approximately 25~33 N of force per square centimeter of muscle cross-sectional area [14]. Considering the area of the muscles, the force measurement capacity of the sensor should have at least a range of 100 N to ensure interaction between the user and the robot. Next, the sensor should be designed robustly. Wearable robots are easily exposed to unexpected situations by users or surrounding environments. Typically, force sensors that have been developed are susceptible to impact and are prone to breakdown. If the sensors suddenly breakdown while the robot is operating, the user is exposed to dangerous situations. To secure the safety of the user and the stability of control, a robust sensor structure is required. The sensor should also be compact and lightweight. Because wearable robots are worn in close contact with the users’ bodies, the space for installing force sensors is limited. Most of the developed force sensors are large or heavy, making them difficult to install on wearable robots, and even if they are installed, they play a role in the wearable robot’s performance degradation. Therefore, it is necessary to develop force sensors with a compact configuration to enable them to be mounted on wearable robots, and such sensors must be developed in a lightweight form to prevent performance degradation of the robot. Finally, it should be easy to manufacture the sensor. To equip wearable robotic systems with multiple force sensors, the cost of manufacturing the sensor should be low. Here, the cost of manufacturing includes not only the price but also the level of manufacturing difficulty. If the sensors applied to wearable robots can be manufactured at reasonable prices, they will contribute greatly to the commercialization of wearable robots.

In this study, we propose a novel sensor based on these features. The proposed sensor is designed to withstand strong interaction forces through the bolt and nut combination. Additionally, the sensor is compact and lightweight. The proposed sensor is manufactured using a simple structure and fabrication process, and most of its components are ready-made products. Therefore, manufacturing sensors is inexpensive. By using the capacitive sensing method proposed by Lee et al. [15], the three-axis force/torque can be measured. The capacitive sensing method, which measures the force/torque through the change in capacitance between electrodes, is used in many applications because the sensor can be compactly manufactured and highly precise [15,16,17,18,19,20]. Therefore, this compact and accurate sensing method is used to measure the three-axis force/torque of the proposed sensor. The capacitive sensing method used in the proposed sensor has many advantages for use in wearable robots when compared to the previously mentioned methods. Capacitive sensors are mechanically simple, and thus, rugged sensors can be easily manufactured. Moreover, because of their simple structure, these sensors are inexpensive. Additionally, they consume low power and are stable with a high resolution and a wide sensing range. However, these sensors have a nonlinear output, and thus, a precise calibration process is required. To measure the force of the artificial muscle of the wearable robots, a sensor was designed in the form of a round loop that can hang the artificial muscle. Thus, a high-load and compact three-axis force sensor was fabricated. The sensor had only four components, and with these components, the assembly process was completed in six steps. The sensor was very small and light, with a diameter of 20 mm, a height of 29 mm, and a weight of 14 g. Next, to evaluate the sensor’s performance, several experimental setups were configured and used in the experiment. First, to convert the raw data of the sensor into force/torque information, we conducted a calibration experiment. We used a deep neural network for accurate calibration. To measure the repeatability of the sensor, we used an experimental setup consisting of a motorized stage, and to measure the capacity of the sensor, we configured an experimental setup that could withstand a load of up to 30 kg. Finally, the developed sensor was applied to artificial muscles, and the performance of the sensor was evaluated.

The rest of this paper is organized as follows: the development of the proposed sensor is introduced in Section 2. The experimental evaluation is explained in Section 3. Finally, the discussion and conclusion are provided in Section 4.

## 2. Development of the Proposed Sensor

### 2.1. Design of the Proposed Sensor

**Figure 1:**
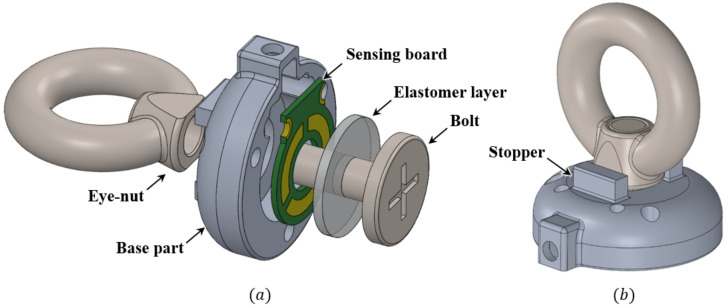
Structure of the proposed sensor. (**a**) Exploded view of the proposed sensor. (**b**) Prototype of the proposed sensor.

The exploded view shown in Figure 1a illustrates the conceptual design of the proposed sensor. The proposed sensor consists of an eye-nut and a bolt, a base part, a sensing board for measuring capacitance, and an elastomer layer. To hang the tendon of the artificial muscles easily, a ring-shaped eye-nut is chosen for the proposed sensor. The bolt that fits the size of this eye-nut is also selected. The base part is designed in a cut hemispherical shape. On its top, rectangular stoppers are designed to restrain the eye-nut’s axis directional rotation. On the sensing board, three sensing cells are printed in a circle around the middle hole of the sensing board to measure three-axis force/torque. To connect the ground of the sensing board and the base part, two semicircular holes are designed on the sensing board, which is connected to the base part using screws. The elastomer layer is then placed between the sensing board and the bolt to create a gap. These five parts are assembled to complete the proposed sensor, as shown in Figure 1b.

**Figure 2:**
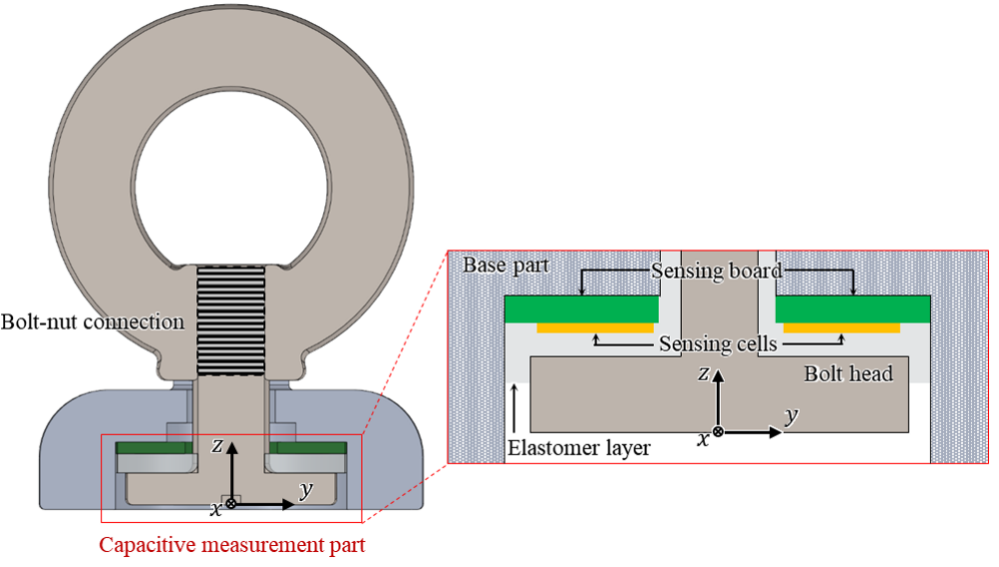
Sectional view and the bolt-nut connection of the proposed sensor.

The sectional view of the assembled proposed sensor is shown in Figure 2. The proposed sensor is designed based on the clamping force that occurs from the connection between the eye-nut and bolt. The robustness and measurement range of the sensor depend on how many external forces the sensor can withstand. Therefore, when designing the sensor, it should be designed to prevent plastic deformation or breakage within the measurement range, taking into account the external forces to which the sensor is exposed and the range of forces being measured. The proposed ring-type sensor is designed based on the connection between the bolt and nut, ensuring the robustness of the sensor and a relatively wide force measurement range. The connection between the bolt and nut used in the ring-type sensor is one of the strongest mechanical combinations most commonly found in everyday life. With the friction of the screw’s threads, this combination can withstand a significantly large force, even though it is very compact. The combination of the M4 bolt and the eye-nut used in the proposed sensor can withstand a force of approximately 688 N [21]. As it is common in everyday life, it is inexpensive and easy to obtain. With this efficient bolt-nut connection, the proposed sensor is designed with a structure that can withstand a large force, even though it is compact and lightweight.

In the proposed sensor, the capacitance measurement part for measuring the three-axis force/torque is configured as shown in Figure 2. It consists of the base part, the sensing board, three sensing cells on the board, the head of the bolt connected to the eye-nut, and the elastomer layer. In this configuration, the capacitance between the three sensing cells on the sensing board and the bolt head acting as a ground is measured. The elastomer layer between the two parts prevents a short circuit and ensures the elasticity of the proposed sensor. The simplicity and compactness of the proposed sensor structure is achieved by this elastomer layer.

### 2.2. Sensing Principle

In the proposed sensor, the force applied to the eye-nut changes the distance between the bolt and the electrodes on the sensing board. These changes result in capacitance variation, which transforms into three-axis force/torque. The capacitance sensing method is used in the proposed sensor to measure the transformed three-axis force/torque. Generally, a capacitive sensor consists of two conductive electrodes and a dielectric substrate between them. The change in capacitance is measured as the distance between these two electrodes changes [17]. The capacitance between two parallel conductors is expressed as follows [16]:

(1) $$C=\varepsilon_{0}\varepsilon_{r}\frac{A}{d},$$

where *C* represents the capacitance between two parallel conductor plates, $\varepsilon_{0}$ denotes the absolute permittivity of free space, such as air, and $\varepsilon_{r}$ denotes the relative permittivity of the dielectric between the plates. *A* represents the area of overlap of the two plates, and *d* represents the separation between the plates. In other words, the important meaning of Equation (1) is that once the two types of permittivity are determined, the capacitance depends only on the overlapping area *A* and thickness *d*. However, in general, the change in the capacitance resulting from the overlapping area *A* of the two plates is very small and the accuracy of the measurement is very low compared to the case where the thickness changes [17]. Therefore, the proposed sensor eliminates this effect through the design of the area of the bolt head, which is a (-) electrode and sufficiently wider than the sensing cells, which are (+) electrodes, on the sensing board. As a result, there would be no overlapping area change. Thus, as the overlapping area does not change, the capacitance changes only as a result of the thickness *d* between the two plates.

**Figure 3:**
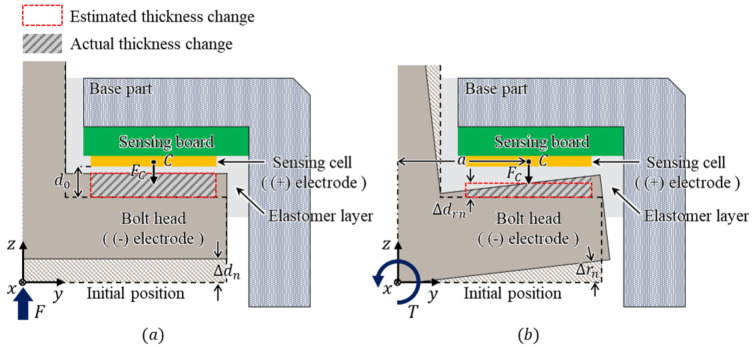
Cross-sectional view of the sensing structure of the proposed sensor. (**a**) Displacement by applied force. (**b**) Displacement by applied torque.

Figure 3 shows an enlarged cross-sectional view of the capacitance measurement part of the proposed sensor. In this configuration, the capacitance between the sensing cell ((+) electrode) and the bolt head ((-) electrode) is measured. Between them, there is an elastomer layer, which is a dielectric substrate. The capacitance between the two plates is changed by the deformation of the elastomer layer. This deformation is caused by external forces, resulting in a thickness change between the two plates. For small strain, when an external force is applied to the sensor, to analyze the tendency of capacitance change, the change in the thickness of the elastomer layer (Δd) can be estimated as follows [22]:

(2) $$\Delta d=\frac{F_{C}}{4BL(E_{h}+ECS_{0}^{2})}d_{0},$$

where $F_{C}$ represents the normal force in the sensing cell for the external force applied to the sensor, $d_{0}$ represents the initial thickness of the elastomer layer, 2B and 2L represent the length and width of the elastomer layer, respectively, $E_{h}$ represents homogeneous compression modulus, *E* represents Young’s modulus of the elastomer layer, $S_{0}$ represents the original shape factor of the elastomer layer, and *C* is constant given by: C=3/4+(2−1.1B/L)B/L.

As shown in Figure 3a, when the force *F* perpendicular to the bolt head of the sensor is applied, the force $F_{C}$ in the sensing cell is applied to the elastomer layer. Accordingly, the bolt head displacement $\Delta d_{n}$ is calculated using Equation (2) in the normal direction. Next, as shown in Figure 3b, when the torque *T* is applied to the bolt head, the normal force $F_{C}$ can be calculated as T/a, where *a* represents the distance from the Z-axis to the center of the sensing cell. In this case, the angular displacement $\Delta r_{n}$ occurs as a result of the applied torque, and the capacitance also changes. The actual thickness change is in the form of a trapezoid. However, it can be estimated as a rectangular form. Therefore, the thickness change can be calculated using Equation (2), in the same way as in the case of the normal direction force [23]. Therefore, the capacitance change is calculated using Equation (1) with the changed thickness *d*, as follows:

(3) $$d=d_{0}-\left(\Delta d_{n}+\Delta d_{rn}\right),$$

where $\Delta d_{rn}$ represents the estimated thickness change as a rectangular form.

**Figure 4:**
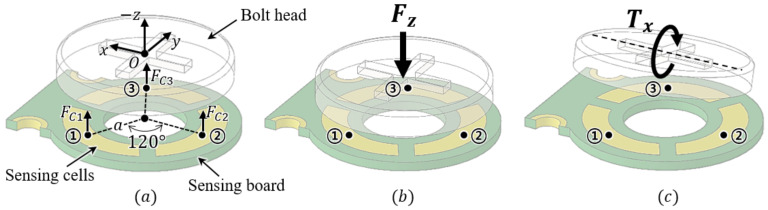
Conceptual view for measuring three-axis force. (**a**) Isometric view of the bolt head and three electrodes. (**b**) Displacement of electrodes when Z-axis force is applied. (**c**) Displacement of electrodes when Y-axis torque is applied.

This concept of capacitance measurement through changes in thickness can be extended to the measurement of the three-axis force/torque, as illustrated in Figure 4. In this figure, to ease understanding, the sensor is reversed to represent the head of the bolt upward. The proposed sensor measures the change in capacitance between the three sensing cells on the sensing board and the head of the bolt, as shown in Figure 4a. The three-axis force/torque can be measured using this proposed structure of the sensing cells [15]. The aspect of changes in capacitance in the sensing cells is related to the change in the size of the gap between the three sensing cells and the head of the bolt, as illustrated in Figure 4b,c. When a force in the direction of the Z-axis is applied to the bolt head, the sizes of all the gaps between the bolt head and the sensing cells change equally depending on the direction of the force, as shown in Figure 4b. Accordingly, the capacitances of the three capacitors change as opposed to the size of the gap. When torque in the direction of the X-axis is applied, the size of the gap between cell 1 and the bolt head changes. At the same time, the size of the gap in cell 3 changes inversely. The overall size of the gap in cell 2 does not change if ideal. Similarly, when torque in the direction of the Y-axis is applied, differences in the sizes of the gaps between cells 1 and 3 and the bolt head have the same aspect, and the aspect of the gap size difference in cell 2 is opposite to that of cells 1 and 3. These relationships are summarized in Table 1.

The three-axis force/torque transformation is derived using the geometrical relations between the normal forces on the sensing cells ($F_{C1}$, $F_{C2}$, $F_{C3}$) and the three-axis force/torque. As depicted in Figure 4b,c, $F_{z}$, $T_{x}$, and $T_{y}$ are expressed as follows:

(4) $$\begin{array}{cc} \; & F_{z}=\left(F_{C1}+F_{C2}+F_{C3}\right)/3 \end{array}$$

(5) $$\begin{array}{cc} \; & T_{x}=-F_{C1}a\cos\left(\pi /6\right)+F_{C3}a\cos\left(\pi /6\right) \end{array}$$

(6) $$\begin{array}{cc} \; & T_{y}=F_{C1}a\sin\left(\pi /6\right)+F_{C3}a\sin\left(\pi /6\right)-F_{C2}a. \end{array}$$

**Figure 5:**
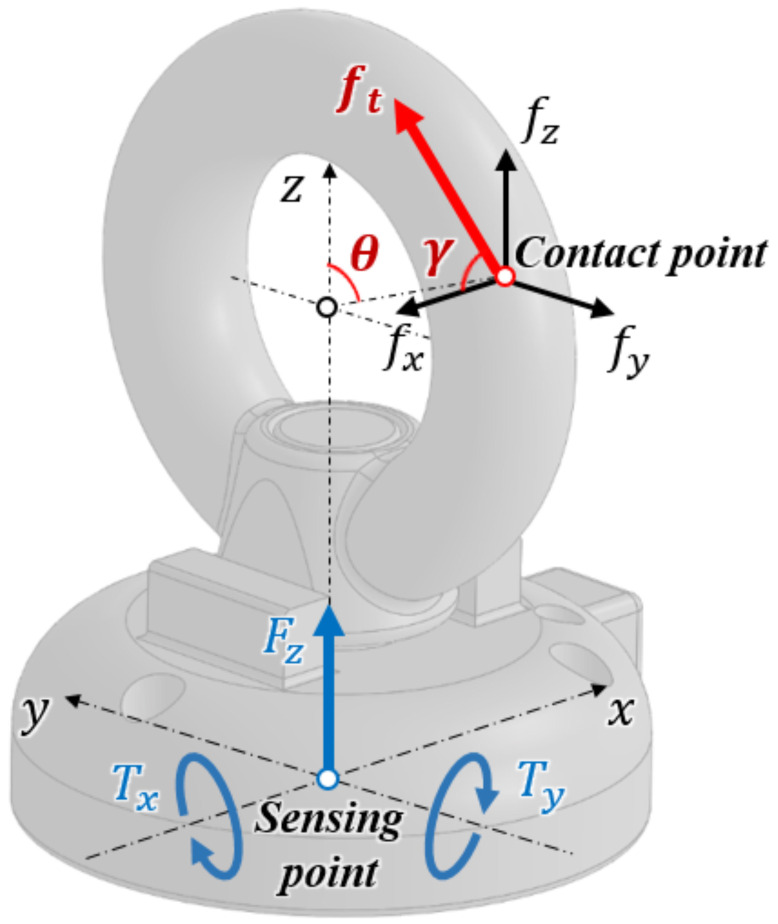
Schematics of the three-axis force/torque element and the decomposed forces applied to the sensor.

As shown in Figure 5, the proposed sensor measures one force ($F_{z}$) and two torques ($T_{x}$, $T_{y}$) at the base of the sensor. When the tendon is hung on the ring of the eye-nut, the tension force ($f_{t}$) is applied to the sensing point. This force can be decomposed into a three-axis force ($f_{x}$, $f_{y}$, $f_{z}$). It is assumed that the distance between the sensing point and the center of the eye-nut’s ring is equal to the outer radius (*r*) of the ring. Therefore, if the rotated angle (θ) of the sensing point can be estimated, the position can also be estimated.

Next, when tension force is applied, assuming that there is no slip on the ring’s surface, the angle between the tension force and the force of the z and y ($f_{z}$, $f_{y}$) axes is constant. Therefore, if the angle (γ) between the force of the X-axis ($f_{x}$) and the tension force can be estimated, the direction of the tension force can also be estimated.

**Figure 6:**
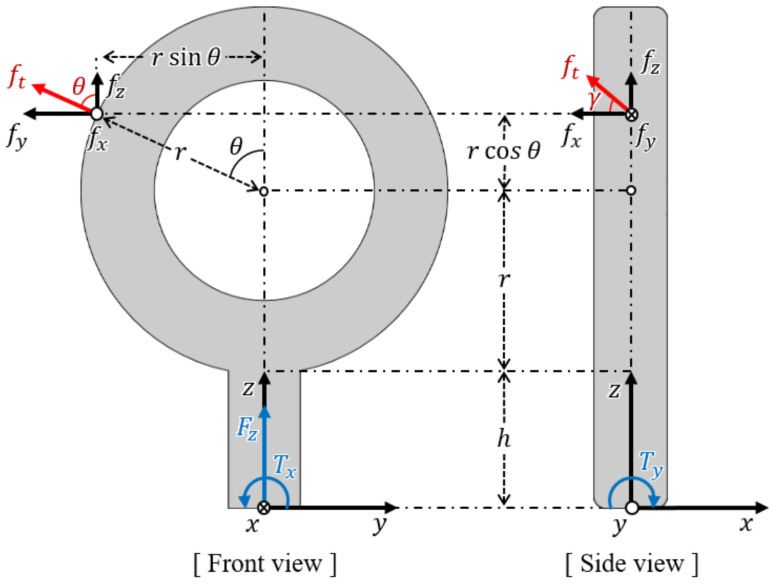
Force and torque configuration with the decomposed forces applied to the eye-nut.

By using the geometric relationship of the sensor, the decomposed three-axis forces can be transformed from the three-axis force/torque measured by the sensor. Figure 6 depicts the geometric relationship between the applied force ($f_{x}$, $f_{y}$, $f_{z}$), measured force, and torques ($F_{z}$, $T_{x}$, $T_{y}$).

From the front view, force and torque ($F_{z}$, $T_{x}$) measured by the sensor and the two decomposed force components ($f_{y}$, $f_{z}$) of the applied force have the following relationship:

(7) $$\begin{array}{cc} \; & F_{z}=f_{z} \end{array}$$

(8) $$\begin{array}{cc} \; & T_{x}=\left(r\cos\theta +r+h\right)f_{y}-r\sin\theta f_{z}, \end{array}$$

where *r* represents the outer radius of the eye-nut’s ring, θ represents the rotated angle of the sensing point, and *h* represents the distance between the bottom of the ring and the base of the sensor. Additionally, from the side view, torque ($T_{y}$) and the decomposed force ($f_{x}$) have the following relationship:

(9) $$T_{y}=-\left(r\cos\theta +r+h\right)f_{x}.$$

The equations above are expressed in the form of a transformation matrix that relates applied decomposed forces to measured force/torque as follows:

(10) $${\left[F_{z}\;T_{x}\;T_{y}\right]}^{T}=T\cdot {\left[f_{x}\;f_{y}\;f_{z}\right]}^{T},$$

where *T* represents the transformation matrix between the measured force/torque and the decomposed forces. From Equations (7)~(10), the inverse matrix of *T* is derived as follows:

(11) $$T^{-1}=\left[\begin{array}{ccc} 0 & 0 & -\frac{1}{h+r+r\cos\theta }\\ \frac{r\sin\theta }{h+r+r\cos\theta } & \frac{1}{h+r+r\cos\theta } & 0\\ 1 & 0 & 0 \end{array}\right].$$

By using this matrix, the measured force/torque can be transformed into three-axis decomposed forces on the eye-nut ring’s surface. As shown in the front view of Figure 6, for the nonslip condition of the ring’s surface, the two forces $f_{y}$ and $f_{z}$ applied to the sensing point have the following relationship:

(12) $$\frac{f_{y}}{f_{z}}=\tan\theta .$$

From the above relationship, the resultant force of the two forces $f_{y}$ and $f_{z}$ exists in line with the radius of the eye-nut’s ring. As a result, from Equations (10) to (12), the rotated angle θ is expressed as follows:

(13) $$\theta =\arctan\frac{T_{x}}{\left(r+h\right)F_{z}}.$$

In Equation (13), $F_{z}$ and $T_{y}$ can be measured by the sensor, and *r* and *h* are designed values. Thus, the rotated angle θ can be calculated. The sensing point of the applied forces is limited to the circumference of the eye-nut’s ring. Therefore, the position of the sensing point is determined.

Next, the angle γ between $f_{t}$ and $f_{x}$ can be calculated as follows:

(14) $$\gamma =\arctan\frac{f_{t}^{\prime }}{f_{x}}.$$

In Equation (14), $f_{t}^{\prime }$ represents the resultant force of $f_{y}$ and $f_{z}$, and $f_{x}$ represents the decomposed X-axis force. These two forces can be calculated using the relationship described above. Thus, the angle γ can also be calculated.

### 2.3. Fabrication Process

**Figure 7:**
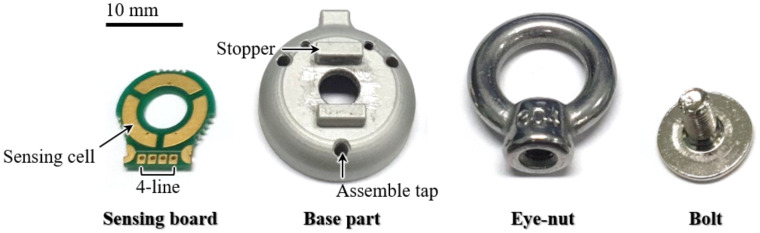
Actual components of the sensor.

As shown in Figure 7, the sensor has only four real components, which are assembled through a simple process. In the sensing board, four lines for communication are configured, and a capacitance-digit-converter (CDC) chip (AD7147, Analog Devices, Wilmington, Massachusetss) is placed on the backside to measure the capacitance of the three sensing cells. The digitized capacitance data from the CDC chip are transferred to the computer through a microcontroller (MCU) chip and a controller area network (CAN) communication bus. The bolt and the eye-nut are ready-made in the size of M4, and the base part is made of aluminum 6061 alloy.

**Figure 8:**
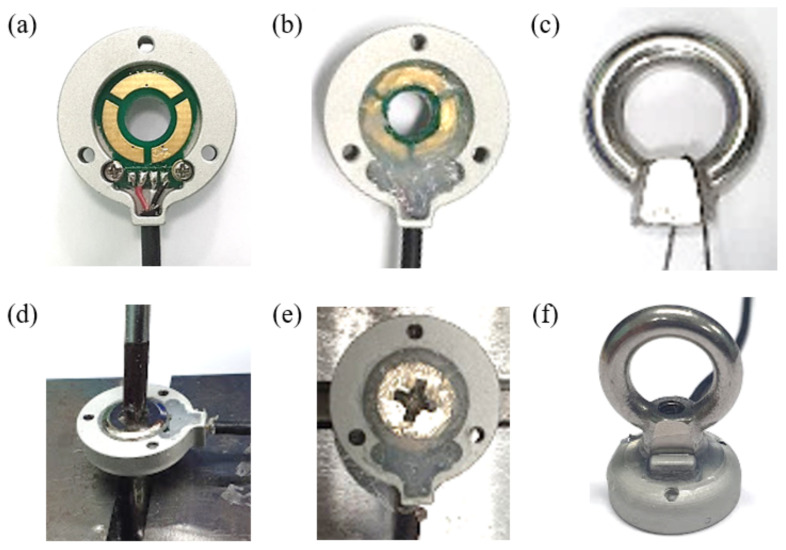
Fabrication process of the developed sensor. (**a**) Connect the sensing board with the base part. (**b**) Add a silicon adhesive. (**c**) Hang a conductive wire. (**d**) Tight with a bolt. (**e**) Coagulate adhesive. (**f**) Fabrication complete.

The sensor is fabricated in six steps using these components, as shown in Figure 8. First, the sensing board part is attached to the base part through two semicircular holes. A silicon adhesive (KE-45T, SHINETSU, Tokyo, Japan) is then applied to create an elastomer layer between the electrode of the sensing board and the bolt. This layer’s thickness is designed to be 0.5 mm. To ground the eye-nut and the bolt to the base part, the conductive wire is inserted into the hole of the eye-nut. The bolt is then inserted into the hole of the sensing board and the base part, and it is tightened to the eye-nut. By using spacers, the size of the gap between the eye-nut and the base part is maintained at 0.5 mm, after which the adhesive between the bolt and the base part is coagulated for approximately 6 h, and the same adhesive is applied to this gap. The sensor is fabricated through the process described above. The fabricated sensor is compact and has a diameter of 20 mm and a height of 29 mm. The sensor has only four main components. Therefore, at 14 g, the sensor is significantly light. In addition, the eye-nut and bolt are common ready-made products, and the sensing board and the base part are cheaply manufactured, so the production of the sensor is inexpensive.

## 3. Experimental Evaluation

### 3.1. Calibration Using Deep Neural Network

**Figure 9:**
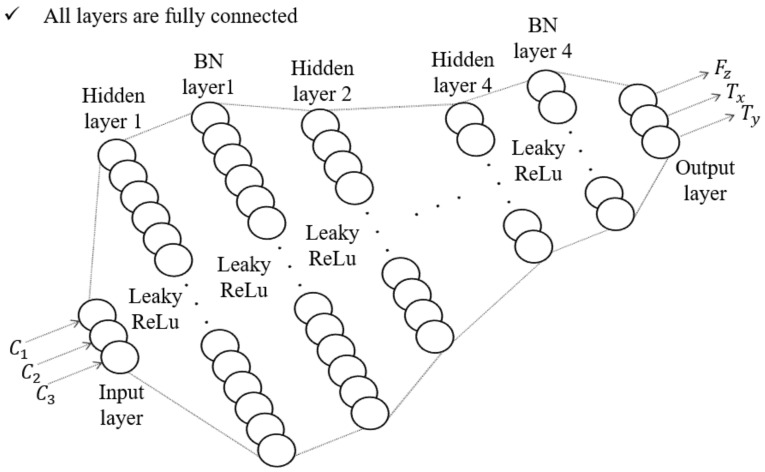
The model of the DNN calibration method.

In general, to obtain accurate results for the sensor’s calibration process, a delicate experimental setup should be designed such that it can apply an independent force and torque to the developed and reference sensors simultaneously. Designing such a sophisticated experimental setup is expensive and time-consuming. Furthermore, this increases the cost of manufacturing sensors. However, through the deep neural network (DNN) calibration method [24], relatively accurate calibration results can be obtained with combined force and torque. In addition, it was assumed that the deformation of the elastomer layer is a linear model in Equation (2). However, actual deformation is too complex by the nonlinearity, hysteresis, and relaxation effect of the elastomer layer. So, it is difficult to predict the change in capacitance with theoretically derived equations. Therefore, to find the relationship between force and capacitance, the DNN calibration method was adopted. Trough this method, the three-axis force/torque could be calculated from the change in the capacitance of the proposed sensor’s cells. As a result, to calibrate the proposed sensor, the DNN model shown in Figure 9 was designed. As shown in Figure 9, the normalized values of the three capacitance variations measured in the cell are input layers, and the sensor’s force and torque values are output layers. Four hidden layers and batch normalization (BN) layers are designed between the input and output layers. There are 180, 90, 60, and 30 number of nodes in each hidden layer. The activation function of the input and output layers is the linear function, and the activation function of the hidden layer is the Leaky ReLu function [25]. The Adam optimizer is used to train this model [26], and the mean squared error is used as the loss function. The train loss and accuracy of this network model are measured as 0.003 and 0.95, respectively.

### 3.2. Experiments

**Figure 10:**
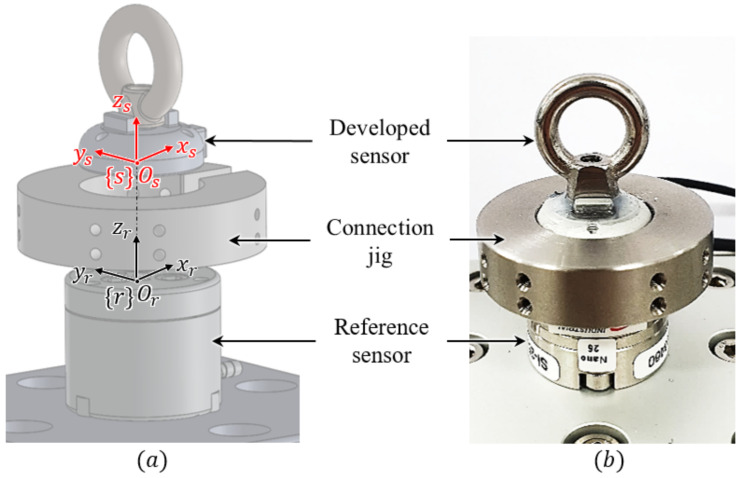
Experimental setup for comparing the three-axis force/torque data measured by the developed sensor with the reference sensor. (**a**) Calibration setup used to obtain the measured data from the developed sensor and the reference sensor. (**b**) Actual assembled state.

**Figure 11:**
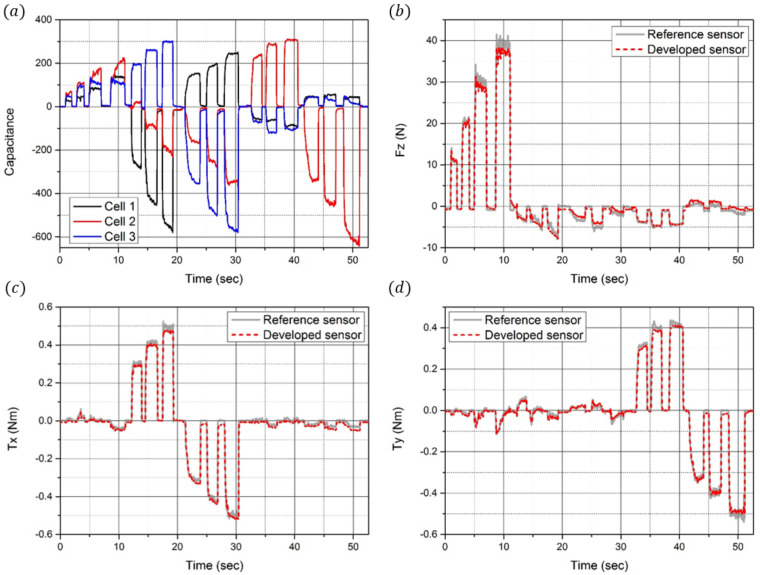
Calibration results. (**a**) Time domain responses of the capacitance change in the three sensing electrodes. (**b**) Time domain response of the z-direction force measured by the developed sensor (red dot) and the reference sensor (gray line). (**c**) Time domain response of the x-direction torque measured by the developed sensor and the reference sensor. (**d**) Time domain response of the y-direction torque measured by the developed sensor and the reference sensor.

For calibration training using the aforementioned DNN method, the reference force and torque data and the raw capacitance data from the developed sensor are required for regression. Therefore, as presented in Figure 9, a calibration setup was designed for calibration. It consists of a reference sensor (F/T Sensor Nano25, ATI Industrial Automation), the developed sensor, and a connection jig to match the axes of the two sensors. As shown in Figure 10a, by matching the reference frames (*s* and *r*) of each sensor, the coincidence force and torque can be applied to the developed sensor and the reference sensor simultaneously. Through the calibration experimental setup shown in Figure 10b, both sensors can measure the same magnitude and direction of the force and torque. The measured capacitance data from the developed sensor were then calibrated using the DNN method. The results of this calibration are shown in Figure 11. Figure 11a shows the datasets of the capacitance variation at the three cells when the force and torque were randomly applied to the eye-nut of the developed sensor for approximately 52 s. With these datasets, the learning results of the DNN model are shown in Figure 11b–d. Each case, in turn, represents the data of $F_{z}$, $T_{x}$, and $T_{y}$, which are data measured at the developed sensor and the reference sensor. In this result, $F_{z}$ is applied for 0~12 s, $T_{x}$ is applied for 12~30 s, and $T_{y}$ is applied for 31~51 s to the sensor.

In this calibration process, precisely independent force and torque were not applied, but complex force and torque were applied. Typically, the independent force and torque are important for calibrating the raw data of the developed sensor in other calibration methods, such as the linear estimation method. In this method, the accuracy of the sensor is ensured when the precisely independent force and torque data can be measured. Thus, in this study, a highly sophisticated experimental setup is required to separate the force and torque of each axis and apply them to the sensor. However, with a relatively simple experimental setup and the proposed DNN calibration method, it is confirmed that the accuracy of the developed sensor is high, as seen in the calibration results. The relative error of the measured force is 2.25%, and the relative errors of the measured torques are 1.71% and 1.64%. Additionally, resolutions of the force and torque were analyzed as 0.25 N, 1.38 m Nm, and 2.47 m Nm. In addition, sensitivities were analyzed as 0.0012 pF/N, 0.22 pF/Nm, and 0.12 pF/Nm. The relative errors, resolutions, and sensitivities are summarized in Table 2.

**Figure 12:**
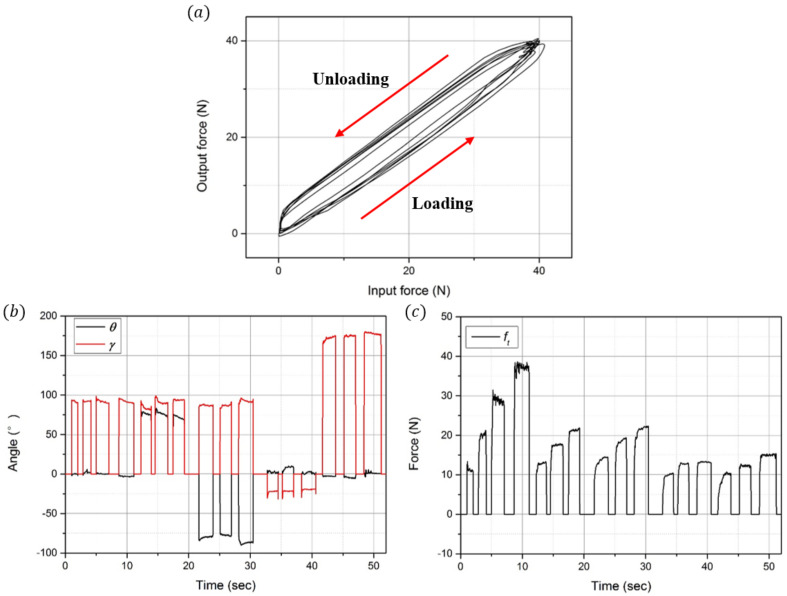
Hysteresis graph and estimation of the angles and magnitude of the applied forces. (**a**) Hysteresis graph of the developed sensor. (**b**) Position angle (θ) and direction angle (γ) of the applied forces. (**c**) Magnitude of the applied forces.

In order to analyze the hysteresis of the developed sensor, a constant force (40 N) was loaded/unloaded five times on the developed sensor in the experimental setup as shown in Figure 10b. As a result, hysteresis was found to be approximately 17.3%. As shown in Figure 12a, while the sensor shows some linearity when loading forces, this tends to decrease slowly when unloading owing to the residual stress in the elastomer layer. As shown in Figure 12b, with the measured data from Figure 11b–d, the two angles (θ, γ) are estimated using Equations (9) and (10). As shown in Figure 12c, the magnitude of the applied forces ($f_{t}$) is calculated as follows: $\left|f_{t}\right|=\sqrt{f_{x}^{2}+f_{y}^{2}+f_{z}^{2}}$.

First, during the 0~12 s when $F_{z}$ is measured, the angle of the sensing point (θ) is approximately 0°, and the angle of the applied force (γ) is approximately 90°. Based on these estimated angles, it can be observed that the force in the z-direction is applied at the top of the ring. Because only $F_{z}$ is applied in this section, the magnitude of the applied force is similar to that of $F_{z}$. Next, during the 13~30 s when $T_{x}$ is measured, θ and γ are approximately 90° when positive $T_{x}$ is measured. Moreover, θ is approximately −90°, and γ is approximately 90° when negative $T_{x}$ is measured. Additionally, it can be observed that the force in the x-direction is applied at the left/right side of the ring. It can also be observed that the magnitude of the applied force increases from 10 N to 20 N. Finally, during the 31~52 s when $T_{y}$ is measured, θ is similar to when $F_{z}$ is measured. However, γ is approximately −10° when positive $T_{y}$ is measured and is 180° when negative $T_{y}$ is measured. It can be observed that the force in the x-direction is applied at the same position as when $F_{z}$ is measured. In addition, the magnitude of the applied force increases from 10 N to 15 N.

**Figure 13:**
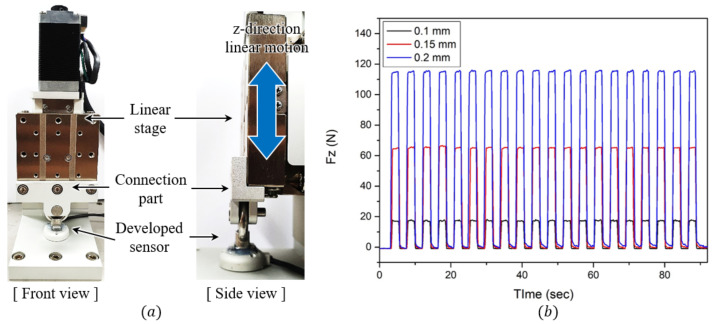
Experiment for checking repeatability. (**a**) Front and side views of the experimental setup configuration. (**b**) Results of the three repeatability experiments.

Next, as shown in Figure 13a, an experimental setup was designed to analyze the force repeatability of the developed sensor. It consists of a z-direction linear motorized stage (ZCV620-F-N, MISUMI), a developed sensor, and a connection part that connects the sensor with the stage, as shown in Figure 13b. In this experimental setup, the manufactured pin was caught between the eye-nut of the sensor and the connection part. Therefore, the linear stage and the developed sensor had the same Z-axis movement, which was measured using $F_{z}$ in the sensor. The linear motorized stage repeatedly moved 20 times at distances of 0.1 mm, 0.15 mm, and 0.2 mm. The time-domain responses of the sensor are shown in Figure 13c. In each case, the percentage errors are expressed based on the average of the measured forces as 1.32%, 1.48%, and 2.31%. As a result, the average repeatability of the force was analyzed as 1.75%. These results are summarized in Table 2.

**Figure 14:**
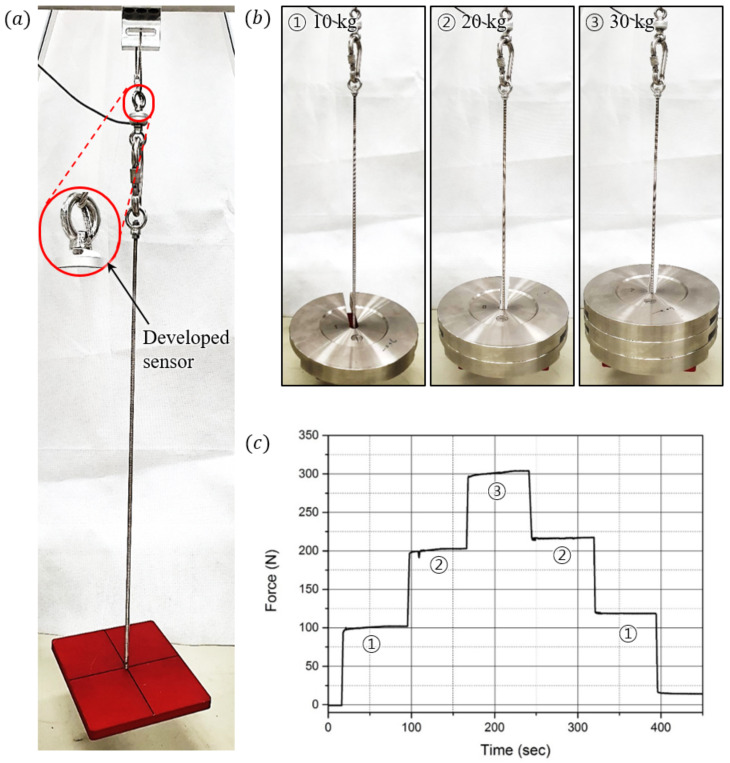
Experiment to evaluate high-load capacity. (**a**) Configuration of the experimental setup. (**b**) Experimental scene when loading mass disks of 10 kg, 20 kg, and 30 kg. (**c**) Result of the high-load capacity experiment.

To analyze the capacity of the developed sensor, the experimental environment shown in Figure 14a was prepared so that the sensor could measure the weight of the mass disks. One mass disk weighs 10 kg, and the sensor measured the weight of a total of three disks in stages. Figure 14c shows the force data measured by the sensor as the disks are increased one by one to reach 30 kg and then reduced again. When loading a mass disk, it can be verified that the sensor measures the force with a relatively small error. However, when unloading a mass disk, it can be observed that the error increases by approximately 10 N. This is as a result of the compression set characteristics of the elastomer layer. When this layer is compressed, its thickness is not fully restored and remains reduced even after the compressive force is completely removed. Therefore, as a result of these characteristics, when the same weight was loaded on and unloaded from the sensor, the size of the gap between the cells and the ground of the sensor was not fully restored and remained reduced.

**Figure 15:**
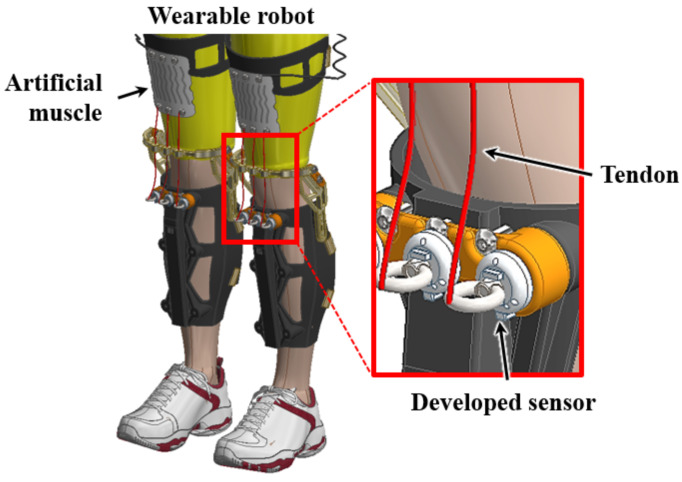
Conceptual assembly modeling of integration of the developed sensor and the wearable robot.

Figure 15 shows the conceptual model of how the developed sensors are integrated into the wearable robot. The base of the sensor is connected to the rigid part of the wearable robot and the artificial muscle is connected to the eye-nut of the sensor through the tendon. Thus, the artificial muscle contracts to assist the wearer’s lower body strength, and the contraction force can be measured by the sensor through the tendon. Thus, the developed sensor was applied to the artificial muscle used in the suit-type wearable robot to measure the contraction force. The artificial muscle based on shape memory alloy springs used in the experimental setup was developed by Park et al. [27,28].

**Figure 16:**
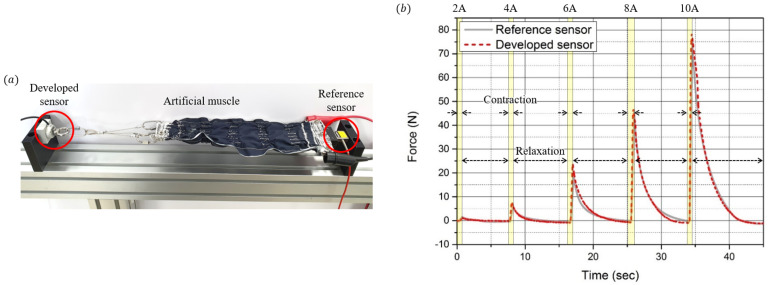
Performance tests with the sensor integrated to an artificial muscle actuator. (**a**) Experimental setup composed of the developed sensor, reference sensor, and an artificial muscle. (**b**) Resulting force of the artificial muscle measured by the developed sensor (red dot) and the reference sensor (black line).

As shown in Figure 16a, the developed sensor and the reference sensor (FSH03878, FUTEK) were applied to both sides of the artificial muscle, and the force of the muscle was measured and analyzed using both sensors. Figure 16b shows the result of the force measured at both sensors. When a current of 2 A, 4 A, 6 A, 8 A, and 10 A is applied to the artificial muscle, the artificial muscle contracts, and when no current is applied, the artificial muscle relaxes. The maximum contraction force of the artificial muscle is approximately 80 N, which is sufficiently measurable using the developed sensor. Comparing the contraction and relaxation phases, it can be seen that a large difference occurs during the relaxation phase. According to the previous hysteresis analysis, the force tends to decrease relatively slowly owing to the residual stress of the elastomer layer when the force is unloaded. The relative error of the reference sensor is 2.37%, similar to the one analyzed earlier. Below a force of 100 N, the compression set characteristic is not manifested. Therefore, the error is smaller than that in the previous high-load experiment.

## 4. Conclusions

In this study, we present a compact, lightweight, and high-capacity three-axis force/torque sensor. The three-axis force/torque sensing is performed using three capacitance sensor cells. The cells were placed in a circular position around the hole on the sensing board. By determining the capacitance variation relationship of the sensing cells and the sensor’s geometric and kinematic relationship, the three-axis force/torque can be derived from the decomposed forces applied to the sensor. Even with its simple structure, the sensor provides three-axis force/torque sensing. The proposed sensor is comprised of only two ready-made parts: one manufactured part and one sensing board. Therefore, the manufacturing and assembly processes are significantly simple. This simple structure of the sensor enables it to be light, compact, and inexpensive. However, despite this simple structure, the bolt-nut tightening force allows the sensor to measure up to a high capacity. We designed a DNN calibration model to transform the capacitance measured in the cells into force/torque data. Through the calibration experiment, we could measure force/torque data by training the DNN model. Next, through several experiments, we evaluated the performance of the sensor. Additionally, we verified the practicality of the sensor by applying it to the developed artificial muscle. The proposed sensor is expected to be used not only in wearable robotics but also in various robotic systems.

## Figures and Tables

**Table 1:** Tendency of capacitance change of three cells for the three-axis force/torque.

		Cell 1	Cell 2	Cell 3
$F_{z}$	+	+	+	+
	-	-	-	-
$T_{x}$	+	-	Δ	+
	-	+	Δ	-
$T_{y}$	+	-	+	-
	-	+	-	+

**Table 2:** Specifications of the sensor.

Quantity	Value	Unit
Sensing range of force	+300	N
Sensing range of torques	±1, ±1	Nm
Resolution of force	0.25	N
Resolution of torques	1.38, 2.47	m Nm
Sensitivity of force	0.0012	pF/N
Sensitivity of torques	0.22, 0.12	pF/Nm
Relative error of force and torque	2.25, 1.71, 1.64	% of FSR *
Average force repeatability	1.75	% of FSR *
Hysteresis of force	17.3	% of FSR *

* FSR: Full-scale force and torque ranges.

## Data Availability

Not applicable.
